# Analysis of Migrant Cyclic PET Oligomers in Olive Oil and Food Simulants Using UHPLC-qTOF-MS

**DOI:** 10.3390/foods12142739

**Published:** 2023-07-19

**Authors:** Dimitra Diamantidou, Emmanouil Tsochatzis, Stavros Kalogiannis, Joao Alberto Lopes, Georgios Theodoridis, Helen Gika

**Affiliations:** 1 Laboratory of Analytical Chemistry, Department of Chemistry, Aristotle University of Thessaloniki, 54124 Thessaloniki, Greece; diamantidou.dim@gmail.com (D.D.); gtheodor@chem.auth.gr (G.T.); 2 Biomic_AUTh, Center for Interdisciplinary Research and Innovation (CIRI-AUTH), Balkan Center, B1.4, Thessaloniki, 10th km Thessaloniki-Thermi Rd, P.O. Box 8318, 57001 Thermi, Greece; 3 Department of Food Science, iFOOD, Centre for Innovative Food Research, Aarhus University, Agro Food Park 48, 8200 Aarhus N, Denmark; emmanouil.tsochatzis@food.au.dk; 4 Department of Nutritional Sciences and Dietetics, International Hellenic University, 57400 Thessaloniki, Greece; 5 European Innovation Council and SMEs Executive Agency (EISMEA), 1210 Brussels, Belgium; joao-filipe.alberto-lopes@ec.europa.eu; 6 School of Medicine, Aristotle University of Thessaloniki, 54124 Thessaloniki, Greece

**Keywords:** cyclic polyester oligomers, QuEChERS clean-up, ultra high-performance liquid chromatography (UHPLC), quadrupole time-of-flight mass spectrometry (qTOF-MS), food contact materials (FCM), migration testing

## Abstract

Oligomers are a particular category of non-intentionally added substances (NIAS) that may be present in food contact materials (FCMs), such as polyethylene terephthalate (PET), and consequently migrate into foods. Here, an ultra-high-pressure liquid chromatography quadruple time-of-flight mass spectrometry (UHPLC-qTOF-MS) method was developed for the analysis of 1st series cyclic PET oligomers in virgin olive oil (VOO) following a QuEChERS clean-up protocol. Oligomer migration was evaluated with two different migration experiments using bottles from virgin and recycled PET: one with VOO samples stored in household conditions for a year and one using the food simulant D2 (95% *v*/*v* ethanol in water) at 60 °C for 10 days. Calibration curves were constructed with fortified VOO samples, with the LOQs ranging from 10 to 50 µg L^−1^ and the recoveries ranging from 86.6 to 113.0%. Results showed no migration of PET oligomers in VOO. However, in the simulated study, significant amounts of all oligomers were detected, with the migration of cyclic PET trimers from recycled bottles being the most abundant. Additional substances were tentatively identified as linear derivatives of PET oligomers. Again, open trimer structures in recycled bottles gave the most significant signals.

## 1. Introduction

Polyethylene terephthalate (PET) is widely used for food and beverage storage due to its advantages, including its thermal and chemical stability, low diffusivity, transparency, and gas barrier properties [1,2,3,4]. During the process of manufacturing PET bottles, some substances can be added to enhance their functional properties and confer some desired characteristics. These substances are EU regulated [5] and known as “intentionally added substances” (IAS). The use of additives helps to enhance or impart specific properties (e.g., plasticizers for flexibility [6,7], antioxidants to avoid polymer oxidation, and flame retardants to impart fire resistance) [8]. Processing aids, such as catalysts, lubricants, and solvents, can enable or ease polymer production and processing [8]. Each step of the polymerization process can generate undesired substances, normally defined as “non-intentionally added substances” (NIAS), which usually include impurities, reaction products such as oligomers, and by- or degradation products. All these chemicals can end up migrating from the polymeric packaging into the food [9,10,11].

PET can be considered a highly inert material as it normally does not require the use of significant amounts of additives, therefore resulting in a low mass transfer of lubricants, plasticizers, and by- or degradation products into foodstuff [12]. Moreover, PET is a packaging material with good recyclability, and thus, PET bottles made of post-consumer recyclates with a content of up to 100% of recycled material are gaining a significant market share [13].

Recycled PET (rPET) is considered to have a decreased risk of containing NIAS due to the limited use of additives during its conversion [11,14]. Its high recyclability and low contamination potential have made rPET the only regulated recycled material that can be used for food packaging in the EU at the moment [11]. The safety of the final product is ensured by following quantitative restrictions on its content or by establishing specific migration limits (SMLs) in mg/kg of additives into food or beverages. According to the EU Plastic Regulation [5], there are SMLs for some of the components used to manufacture PET bottles, such as mono- and diethylene glycol, terephthalic acid, and isophthalic acid. However, currently, no SMLs for PET oligomers, either cyclic or linear, are in place.

The existing literature on the study of oligomer migration from PET food contact materials (FCMs) toward food matrices is still rather limited [1,4,10,15,16,17,18]. Xu et al. [17] used ultra-high-performance liquid chromatography coupled with quadrupole time-of-flight mass spectrometry (UHPLC-qTOF-MS) to assess the concentrations of cyclic PET and polybutylene terephthalate (PBT) that migrated from laminated steel cans in four types of food simulants (3% *w*/*v* acetic acid, 10% *v*/*v* ethanol, 50% *v*/*v* ethanol, and isooctane). The results showed that the amount of oligomers that migrated into isooctane was the most abundant, followed by 50% *v*/*v* ethanol. Ubeda et al. [2] determined the oligomer profile of virgin and recycled PET pellets with UHPLC-qTOF-MS analysis using three simulants (3% *w*/*v* acetic acid, 10% *v*/*v* ethanol, and 95% *v*/*v* ethanol). Again, the official food simulant for fatty foods (95% *v*/*v* ethanol) gave the highest oligomer concentrations, with the cyclic and linear dimers and trimers being the most abundant. However, these previous studies were carried out using food simulants and not real food matrices. By using a modified QuEChERS clean-up protocol, Tsochatzis et al. [10] quantified a total of 11 cyclic oligomers (PET, PBT, and polyester urethane-based (PU)) in pasta samples. In another work, Franz and Welle [18] studied the potential migration of oligomers from PET bottles into food simulants (10% *v*/*v* ethanol, 50% *v*/*v* ethanol and 95% *v*/*v* ethanol) and real beverages (fruit juices and soft drinks). It was found that the oligomer migration levels from PET FCMs were the highest for the 95% *v*/*v* ethanol food simulant. Thus, it seems that PET oligomers are more likely to migrate into foods with high fat content due to their lipophilic nature; yet, there is a lack of studies for their quantification in those food matrices in the current literature.

Herein, we have aimed to study the migration of these species in olive oil, which is expected, based on the above, to be noticeable. Thus, a UHPLC-MS method was developed and validated for this purpose. Due to the complexity of matrices with high lipid contents, such as edible oils, sample pre-treatment for the isolation of PET oligomers is challenging. Many efforts have been made in recent years to improve clean-up steps in such matrices [19,20], mainly involving solid phase extraction (SPE) [21,22] and dispersive solid phase extraction (d-SPE) techniques [20,23]. In the current work, a d-SPE (or QuEChERS) sample preparation method was applied to facilitate analytes’ extraction and sample clean-up, as it was found to be the optimum for the analysis of the four oligomers in oil. To the best of the authors’ knowledge, the application of d-SPE for the analysis of PET oligomers in liquid food samples has not been reported so far. Therefore, the major objective of the present study was to assess the migration of oligomers from virgin and recycled PET bottles into virgin olive oil (VOO) samples and official food simulants based on the developed method. Regarding the migration experiment, two procedures were performed and compared: (1) test conditions according to EU Regulation No. 10/2011 and (2) mimicking the storage of VOO in household conditions. To the best of our knowledge, this study represents the first method for the determination of cyclic PET oligomer migration from PET bottles to VOO under real household conditions and the first fully quantitative method for the analysis of PET oligomers from recycled bottles.

## 2. Materials and Methods

### 2.1. Reagents and Materials

Ethanol (EtOH, HPLC grade ≥ 99.0%) and 1,1,1,3,3,3-hexafluoro-2-propanol (HFIP) (Chromasolv grade) were acquired from Sigma Aldrich (Darmstadt, Germany). Acetonitrile (ACN, LC-MS grade) was supplied from VWR Chemicals (Darmstadt, Germany). Magnesium sulfate (MgSO_4_), silica, and primary secondary amine (PSA) were supplied by Merck (Darmstadt, Germany). Water was purified in a Milli-Q purification system (Merck Darmstadt, Germany) with a resistivity of 18.2 MΩ-cm (at 25 °C). The first-series cyclic PET dimers, trimers, tetramers, and pentamers and the internal standard (IS) cyclic PET trimer-d_12_ were supplied from TRC Chemicals (North York, ON, Canada), with stated purities ranging from 95% to 97%. Blank VOO was obtained from experimental farming, and plastics were avoided in the olive oil production and collection processs.

Only glass labware or containers (e.g., Eppendorf tubes) and syringes made of plastics other than PET (polyethylene and polypropylene) were used in order to avoid any potential source of contamination.

### 2.2. Calibration and Preparation of Standard Solutions

Stock solutions containing 5000 mg L^−1^ of each of the PET oligomers (dimer to pentamer) and the IS were prepared using HFIP as the solvent. Multi-component stock solutions were prepared in amber vials to prevent photodegradation or isomeric conversion [10,24,25,26] and were kept at −20 °C. Appropriate working standard solutions were produced by dilution with EtOH:H_2_O 50:50 (*v*/*v*) containing 5% *v*/*v* HFIP every week at ten concentrations (5, 10, 25, 50, 100, 250, 500, 750, 1000, 2500 µg L^−1^). Calibration curves were constructed by plotting the means of ratios of compound peak areas to the IS peak areas against the analytes’ concentrations.

The evaluations of the calibration, recovery, and matrix effects were performed using fortified olive oil samples. Aliquots of VOO samples were fortified with the IS and known standard mixtures of the 4 cyclic PET oligomers, giving final concentrations of 10, 25, 50, 100, 250, 500, 750, 1000, 1500, and 2500 µg L^−1^. A blank sample was prepared with the same procedure without the addition of a standard.

### 2.3. Migration Tests

Four different 0.5 L PET bottles intended for contact with beverages and edible oils were supplied from a local market. Two of them were from 100% virgin PET (vPET), and the other two were from 100% rPET. All beverage bottles had not previously been in contact with food.

In the first test, the migration experiment was performed according to EU Reg. No. 10/2011 [5]. For fatty foods, compliance testing should be carried out using simulant D2 (vegetable oil) or, alternatively, with isooctane or 95% *v*/*v* ethanol in water. The latter was selected as the simulant to substitute for the use of vegetable oil. Then, both the vPET and the rPET plastic bottles containing the food simulants were stored in an incubator at a temperature of 60 °C for 10 days. Each sample volume was adjusted to 400 mL, and two replicates were prepared. Test times and temperatures were selected to cover a storage period of more than 6 months at room temperature or below, mimicking potential household conditions. After the migration period, 200 µL of each sample was transferred into an LC vial prior to analysis. For the quantification of the less-abundant PET tetramer and PET pentamer, a volume of 2 mL was collected and evaporated to dryness in an Eppendorf concentrator at room temperature. Finally, the sample was reconstituted with 200 µL EtOH:H_2_O 50:50 (*v*/*v*) containing 5% *v*/*v* HFIP, and 5 µL was injected into the LC system.

Time and temperature storage conditions representative of household conditions/real life were selected to measure oligomer migration in real olive oil samples. Thus, in this second migration experiment, PET bottles were filled with 400 mL of “blank” VOO sample (see Section 2.1). Since light and temperature are crucial factors of VOO storing [27], the bottles were sealed and stored at constant room temperature avoiding light exposure. Two replicates for each type of PET bottle (virgin and recycled) were prepared and stored for 12 months at room temperature (~20 °C). Samples were collected at three time points (2, 6 and 12 months) and were analyzed as described in Section 2.4.

### 2.4. Extraction and QuEChERS Clean-Up

VOO samples were prepared using a QuEChERS extraction and a clean-up protocol. Briefly, a 300 mg olive oil sample was placed in a 2 mL Eppendorf tube, to which 100 µg of anhydrous MgSO_4_ had been previously added. The extraction process was conducted by adding 600 µL of ACN:HFIP, 98:2 (*v*/*v*), and the tube was then vortexed for 30 min and centrifuged at 10,000 rpm for 10 min. A freeze-out step to reduce lipid coextracts occurred at −20 °C for 1.5 h. The supernatant was collected and placed in a 1.5 mL Eppendorf tube containing 25 mg of silica. Using the aforementioned prepared mixtures, PSA sorbent and PSA in combination with silica were also tested. After vortexing for 10 min and centrifuging at 10,000 rpm for another 10 min, a volume of 500 µL from the extract layer was transferred to a clean 1.5 mL Eppendorf tube and evaporated to dryness at room temperature using an Eppendorf concentrator. Finally, the sample was reconstituted with 200 µL EtOH:H_2_O 50:50 (*v*/*v*) containing 5% *v*/*v* HFIP. It was then filtered through a 0.22 µm PTFE filter, and 5 µL of the sample was injected into the chromatographic system.

### 2.5. Instrumentation

#### UHPLC-qTOF-MS Analysis

In a previous work from our group [28], we established a UHPLC-qTOF-MS method for the quantification of seven cyclic PET and PBT oligomers in blood. This method was applied here for the analysis of olive oil extracts. In brief, a Waters BEH C18 (150 × 2.1 mm, 1.7 µm) column was used on an Elute UHPLC system (Bruker, Bremen, Germany), operating through a gradient of solvent B (ACN, 0.1% formic acid) versus A (H_2_O, 0.1% formic acid). A TOF mass spectrometer (Bruker, Germany) was operated in positive ionization mode with an ESI source. Full-scan MS data were acquired over a *m*/*z* range of 300–1000 Da at a rate of 3 spectra/min. The capillary voltage was 3.5 kV, dry gas flow was 10 L/min, nebulizer pressure was 2.0 bar, source dry temperature was 300 °C, funnel 1 RF was 200.0 Vpp, funnel 2 RF was 200.0 Vpp, multiple RF were 200.0 Vpp, deflection delta was 60.0 V, transfer time was 100.0 µs, and collision RF was 800.0 Vpp. The individual recalibration of each chromatogram was achieved by injecting sodium formate solution 0.1 to 0.3 min before every analysis. Quantitation was based on the peak areas of selected extracted ions (*m*/*z*), which were as follows: 385.0918, 577.1346, 769.1764, and 961.2192. Compass HyStar 6.0 software (Bruker, Germany) and Data Analysis 5.3 software (Bruker, Germany) were used for data acquisition and handling.

### 2.6. Extraction Recovery and Matrix Effect

The extraction recovery was determined with fortified samples in three concentration levels and was expressed as a percentage calculated using the following equation:

(1) $$R\%\;=\;100\;\times \;\left(\frac{\mathrm{spiked}\;(b)}{\mathrm{spiked}\;(a)}\right)$$

where spiked (b) is the concentration in the olive oil sample before the extraction and spiked (a) is the nominal concentration in the extracts.

The matrix effect (ME) is an important parameter in mass spectrometry since coeluting endogenous compounds could interfere with the analysis via signal suppression or enhancement. Thus, Student’s *t*-test was adopted for ME determination [29] and was calculated according to the equation

(2) $$t\;=\;\frac{b_{1}-b_{2}}{s_{b_{1}-b_{2}}}$$

where b_1_ and b_2_ are the slopes of the regression lines. The standard error of the difference between the regression slopes can be calculated as

(3) $$s_{b_{1}-b_{2}}\;=\;{\left[\frac{{\left(s_{y\cdot x}^{2}\right)}_{p}}{\sum x_{1}^{2}}-\frac{{\left(s_{y\cdot x}^{2}\right)}_{p}}{\sum x_{2}^{2}}\right]}^{\frac{1}{2}}$$

where ${\left(s_{y\cdot x}^{2}\right)}_{p}$ refers to the pool residual mean square, and the subscripts 1 and 2 are the two regression lines (matrix and solvent) when compared. The critical *t*-test values were calculated with (n_1_ − 2) + (n_2_ − 2) degrees of freedom.

### 2.7. Method Validation

The proposed method for the olive oil analysis was validated in terms of linearity, precision, accuracy, and limit of detection (LOD) and quantification (LOQ), according to the Eurachem guide [30]. Linearity was assessed by analyzing spiked samples over the range of 10–2500 µg L^−1^. Calibration functions and coefficients of determination were calculated using linear least squares regression. The LOQs were defined as the lowest concentrations that could be determined with acceptable performance, and the LODs were calculated as 0.33 times the LOQs. Precision and accuracy were assessed with spiked samples by calculating the relative standard deviation (RSD, %) in the short-term (repeatability, intra-day accuracy) and for three consecutive days (intermediate precision, inter-day precision). The measurement accuracy was expressed as the relative error (Er%).

## 3. Results and Discussion

### 3.1. Method Development

#### Optimization of Sample Preparation

The extraction process was evaluated using different solvents (ACN, EtOH:H_2_O, 50:50 (*v*/*v*), and H_2_O), with ACN showing the most satisfactory results (Table 1). Next, the HFIP solvent ratio was also assessed (0% *v*/*v*, 2% *v*/*v*, 5% *v*/*v* and 15% *v*/*v*). Multiple extractions and different HFIP solvent ratios showed no significant difference in extraction recoveries. Three different dilution solvents were tested to evaluate the extraction recovery (dichloromethane, hexane and ethyl acetate). However, none of them was chosen for further experiments. After centrifugation, different sorbents were used for the clean-up step, including PSA, silica, and PSA:silica, 1:1 (*w*/*w*), with each of them in combination with MgSO_4_ to minimize interactions or potential oligomer hydrolysis during analysis with potential residual H_2_O content. All the aforementioned experiments were applied in spiked VOO samples, and the results were evaluated based on the recoveries of the known-spiking levels. According to these results, no significant differences were observed between the three clean-up solvents. Thus, silica was finally selected for the clean-up protocol of olive oil in the presence of 100 mg of anhydrous MgSO_4_. Extraction recoveries for all studied oligomers at three concentration levels (50, 500, and 1000 µg L^−1^) were calculated with Equation (1) and were found to be between 86.6 and 113.0%, which can be considered satisfactory.

Lipid co-extracts may interfere with the chromatographic analysis, causing clogging of the analytical column [31] or ion suppression/enhancement. Both PSA and silica are used to remove triglycerides or fatty acids, among other components; however, in the case of high-fat-content matrices, such as edible oils, a freeze-out step can further improve the removal of co-extracted fats. Thus, along with the optimized protocol, samples were subjected to a freeze-out step at −20 °C for at least 1.5 h. The selection of a non-polar solvent (hexane, dichloromethane, or ethyl acetate) can improve the extraction efficiency of oligomers through the effective dilution of olive oil and the decrease in viscosity. However, a freeze-out step cannot be applied in the presence of such solvents due to their low freezing points; thus, non-polar compounds cannot be effectively cleaned up. Consequently, none of the dilution solvents was selected for the proposed method to enable the application of a freeze-out step.

The effect of the matrix was determined by applying Student’s *t*-test, as described in Section 2.7. The results are summarized in Table 2. Clearly, a statistically relevant difference (at the 95% confidence level) occurred between the calibration curves in the matrix and solvent for all oligomers. Therefore, the spiked calibration curves were used for oligomer quantification to compensate for the bias due to the matrix effect.

**Figure 1:**
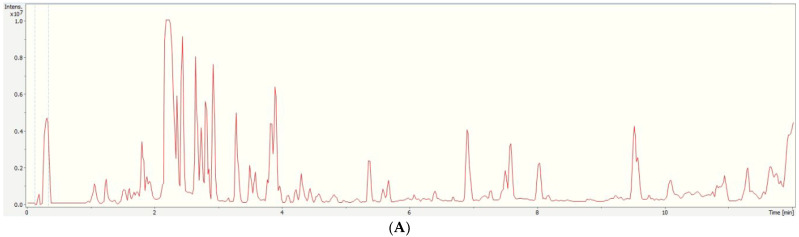

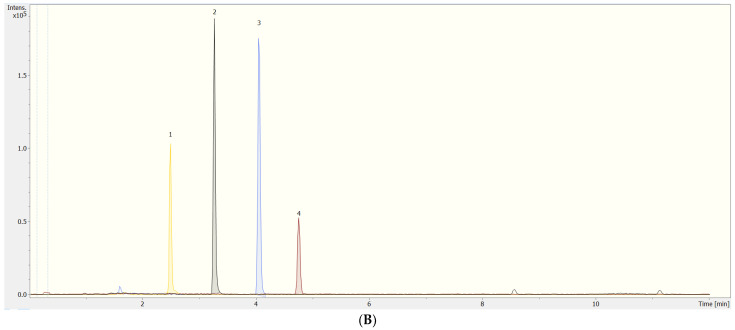
UHPLC-qTOF-MS (**A**) total ion chromatogram (TIC) and (**B**) extracted ion chromatograms (XICs) from a representative VOO sample spiked with a standard multi-component mixture (1500 µg L^−1^) of the targets, including (1) PET dimers, (2) PET trimers, (3) PET tetramers, and (4) PET pentamers. Dashed lines from 0.1 to 0.3 min represent the sodium formate solution for recalibration purposes.

Figure 1A is representative of a highly fortified VOO sample, highlighting the importance of suitable extraction and sample clean-up procedures. The major constituents in olive oil are lipids, which contain fatty acids [32]. Olive oil also contains a considerable amount of phenolic compounds, and their composition mainly depends on production and storage methods [33,34]. Thus, despite the clean-up step, we were able to detect high concentrations of endogenous compounds, mainly co-extracted fatty acids and lipids. Furthermore, organic acids known for their presence in olive oil were also tentatively identified. The identification was based on precursor ions and source fragments, and all mass spectra were compared with the features in the Human Metabolome Database (HMDB) for the specific compounds [35]. Individual chromatograms and mass spectra representing these compounds were manually extracted (Extracted Ion Chromatogram, EIC) after peak integration using Data Analysis 5.3 software (Bruker, Germany).

### 3.2. Method Validation

#### 3.2.1. Linearity and Sensitivity

The linearity, LODs and LOQs were determined to evaluate the method performance and, together with other analytical figures of merit, are presented in Table 3. Calibration curves in fortified VOO samples exhibited linearity between 10 and 2500 µg L^−1^, with coefficients of determination (R^2^) higher than 0.99 in all cases. The LODs ranged between 3.3 and 16.7 µg L^−1,^ and the LOQs ranged between 10.0 and 50.0 µg L^−1^. Since no other studies reporting PET oligomer quantification in olive oil samples are available in the literature, the comparison of the results was based only on reported methods in food simulants [16,18]. However, it is noteworthy that our method presented comparable LODs to those reported previously for the determination of PET oligomers in pasta samples [10].

Especially for PET dimers, we can observe an increased LOD compared to the other tested cyclic oligomers. The same was reported before [10], where the PET dimers presented significantly higher LODs than all the other oligomers. This effect seems to be correlated with the fact that PET dimers are susceptible to a ring-opening mechanism, which may occur during analysis (hydrolysis) in the presence of acidified H_2_O in the mobile phase. The latter effect was indicated and highlighted in a previous work where a cyclic PET dimer was reported to be more sensitive to hydrolysis at temperatures above 4 °C [16,25].

#### 3.2.2. Precision and Accuracy

The precision of the proposed method, expressed as relative standard deviation (S_r_), was below 14.9% for both short-term repeatability and intermediate precision. For the assessment of the method’s accuracy, the relative error was calculated (percentage difference of the nominal value, E_r_%) and found to range from −15.5 up to 17.9% for intra-day error and between −10.1 and 18.4% for inter-day error. All the results for precision and accuracy are presented in Table 4.

### 3.3. Migration of Oligomers in Food Simulants and Olive Oil

The proposed method has been used for the analysis of the target compounds that migrated from vPET and rPET bottles into both food simulants and VOO samples, with the latter being assessed after their storage for 10 days at 60 °C and for 12 months at room temperature. The results showed that there is no PET migration in VOO samples under household conditions after a year of storage. On the other hand, cyclic oligomers were quantified in 95% ethanol simulant after their storage in both types of tested PET bottles, with concentrations ranging from 38.8 to 198 µg L^−1^ for PET dimers, from 587 to 2950 µg L^−1^ for PET trimers, and between 0.51 and 9.32 µg L^−1^ for PET tetramers. Cyclic PET pentamers were only detected in food simulants after migration testing with rPET bottles at concentrations of 2.14 µg L^−1^ and 2.57 µg L^−1^ for the two replicates. The results of the analysis are given in Table 5.

In addition to the cyclic oligomers that were thoroughly identified and quantified by means of standards, in the case of migration testing with food simulants, additional substances were detected and tentatively identified as linear derivatives of cyclic PET oligomers based on their monoisotopic molecular masses. In particular, the open first-series linear PET dimers H-[TPA-EG]_2_-OH (*m*/*z* 403.1016) and H-[TPA-EG]_2_-H (*m*/*z* 387.1924), the trimer H-[TPA-EG]_3_-OH (*m*/*z* 594.1593), as well as the open hydrolyzed second-series trimer H-[TPA-EG]_3_-EG-OH (*m*/*z* 639.1697) were detected in accordance to Tsochatzis et al. [16] as a result of migration from both rPET and vPET bottles (Table S1). However, it is unknown whether they were in this form in the PET matrix or if they were produced from already-migrated cyclic PET oligomers that reacted to open PET derivatives, e.g., via hydrolytic reactions. The latter hypothesis is further supported by the fact that an additional substance giving rise to a significant signal in MS was identified as H-[TPA-EG]_3_-O-C_2_H_5_ (*m*/*z* 623.1744) (Figure S1), which seems to be the product of the transesterification reaction of a cyclic PET trimer with ethanol, also known as ethanolysis. To our knowledge, this is the first time that this product has been detected in a migration study. A tentative quantification of the PET oligomer derivatives, based on the respective calibration curves of the cyclic oligomers, is presented in Table S1. The concentrations of H-[TPA-EG]_2_-OH, H-[TPA-EG]_2_-H, and H-[TPA-EG]_3_-EG-OH are quantified below 115 μg L^−1^ in both types of PET, whereas the concentrations of H-[TPA-EG]_3_-OH and H-[TPA-EG]_3_-O-C_2_H_5_ are in the ranges of 2845–4284 μg L^−1^ in recycled material and 378.7–822.4 μg L^−1^ in vPET, indicating higher migration from vPET, as was the case of cyclic oligomers. The fact that the products of hydrolysis and ethanolysis seem to be present in significant levels could indicate that the true quantities of the originally migrating cyclic oligomers are considerably higher than those found due to their deterioration by further reactions, such as transesterification, occurring after diffusion into the solvent. This becomes of interest in the case of vegetable oils, which are comprised vastly of triglycerides and therefore incapable of undergoing similar reactions with the ester bonds of the cyclic oligomers, hence remaining intact. Our findings are again in accordance with Tsochatzis et al. [16] regarding the linear trimers, although the studied materials (PET teabags vs. PET bottles) and the solvent-to-volume (S/V) ratio were significantly different (Table S1).

Accelerated simulation tests have been designed in order to obtain migration comparable to the worst-case storage scenario at the end of a product’s shelf life [13]. However, Gehring and Welle [36] showed that the test conditions of 10 days at 60 °C in PET led to an overestimation of the migration of larger molecules with higher activation energies of diffusion, such as the PET oligomers. Our results, which show considerable concentration differences between household conditions and the simulation, are consistent with previous studies, which concluded that similar testing conditions of 10 days at 60 °C strongly overestimate the migration under room temperature conditions [13,18,36]. This overestimation can be potentially explained by the swelling effect that occured in PET bottles during the experiment, caused by the interaction between EtOH and the polar PET matrix [18]. Such severe contact conditions seem to accelerate the mass transfer of the oligomers from the material into the simulant or food matrix, which results in the overestimation mentioned above. Furthermore, it was observed that the cyclic PET trimer was the most dominant migrated oligomer, followed by the cyclic PET dimer, a result which is also in accordance with previous findings [2,10,18].

As expected, according to the existing literature, a significant amount of cyclic PET trimers was released from both types of PET bottles. However, migration from rPET was more than doubled compared to vPET bottles. Cyclic PET pentamers were only detected in 95% ethanol simulant in contact with rPET, corroborating the higher oligomer release in comparison to PET bottles. These findings are in disagreement with the study of Ubeda et al. [2], in which no differences between vPET and rPET were observed. This disagreement could be attributed to differences in the analytical and migration test methodologies employed, since our study presents a fully quantitative method employing real analytical standards following the officially recommended testing conditions, which represent the worst-case foreseeable use and storage [5]. However, it also underlines that PET recycling technology could affect the quality of the produced PET regarding minor constituents and NIAS that might have an impact on food safety. All cyclic PET oligomers are of the Cramer III toxicity class [16], and their risk assessment is generally based on the toxicological threshold of 50 µg kg^−1^ for total oligomer migration for new polyester co-monomers FCMs [37], which can be used but have not been officially established for any type of oligomers.

Following the toxicological threshold of concern (TTC) approach, the migration testing results indicated that the PET dimers and trimers might be considered health concerns. Furthermore, the migration of cyclic PET tetramers and pentamers, although of high molar mass, might also be considered health concerns, although no toxic effects have been reported yet.

However, if we consider the accumulated daily exposure to the sum of oligomers, based on the EFSA threshold of 50.0 µg kg^−1^ for total oligomer migration [15,16], all samples of PET bottles, either virgin or recycled, presented significantly higher concentration values of the aforementioned limit. Hence, in this case, if the total amount of migrated cyclic PET oligomers is considered, then all samples are non-compliant. In addition, the latter scenario is a rather conservative one since only the cyclic oligomers were quantified without also considering the respective linear oligomers that can be potentially present. Nevertheless, we need to keep in mind that migration testing represents the worst-case scenario with respect to the FCM’s intended use. In some cases, it is considered to overestimate migration levels of compounds when compared to real-life intended use and storage conditions.

In the case of VOO, none of the aforementioned linear oligomers have been identified, which may indicate the absence of the respective cyclic PET oligomers. Thus, a correlation can be extrapolated between the presence of both cyclic and linear oligomers simultaneously in VOO, due to the migration conditions (absence of water), where no hydrolysis phenomena could have taken place.

Considerable differences in cyclic oligomer migration between VOO stored at household conditions and the simulation test were observed. In VOO, no migration was evident whereas in the simulation test, the concentrations found exceeded the TTC multiple times. Such differences can hardly be attributed to the fact that the simulation test aims to resemble worst-case conditions; on the contrary, they seem to confirm the concerns previously expressed regarding the suitability of the test on PET [13,18,36]. Moreover, we provided evidence that ethanol may not be a suitable substitute for lipids since it can participate in transesterification reactions with cyclic PET trimers.

### 3.4. Oligomers in Virgin and Recycled PET

It can be seen from the test results that the migrating oligomeric content was much higher in the food simulants in the case of the rPET compared with the vPET bottles. More specifically, PET dimer migration was almost 5 times higher in recycled bottles, while PET tetramers reached a 9 times higher migration. An interesting outcome is the detection and quantification of high MW oligomers, such as the PET tetramer (768.12 Da) in both vPET and rPET migration solutions, while the PET pentamer (960.84 Da) was detected only in rPET bottles. This result is reported for the first time, following a fully quantitative methodology. Representative TICs of the food simulant D2 from vPET and rPET are illustrated in Figure S2.

It must be highlighted that there was no available information concerning the recycling and decontamination technology applied to produce the rPET bottles used in this study. That information is also missing in all the works published so far dealing with a vPET and rPET FCM comparison. According to the EFSA publication repository, a significant number of different technologies can be applied for the decontamination and recycling of food contact PET, such as Starlinger iV + technology [37], Vacurema Prime [38], EREMA Basic [39], and Kreyenborg IR Clean+ [40]. Throughout these technologies, different decontamination conditions, extrusion steps (single screw vs. twin screw), single or reactor combinations, solid-state polycondensation (SSP) techniques and infrared (IR) technologies are applied. In addition, all these technologies follow different operating conditions, such as heating, pressure, and extrusion, with unknown effects on the generation/formation of cyclic PET oligomers. Thus, no knowledge exists at the moment to indicate to what extent the process, per se, can potentially lead to the reduction or the de novo formation of cyclic PET oligomers. Hence, it is unknown whether the vPET bottles used in the present study and in the study of Ubeda et al. [2] used different recycling technologies, which could also be causes for the reported differences in migration. It is well known that a low percentage of cyclic PET oligomers can exist in PET from its synthesis onward. Even in cyclic-oligomer-free PET, the formation of cyclic oligomers from the melt might occur at a significant level, thus re-increasing the cyclic oligomer content. In this case, the fraction of the re-formed cyclic oligomers showed that a majority of cyclic trimers (60–70%) was found at equilibrium [41].

Therefore, the results of the present study seem to indicate that the formation of oligomers may be correlated with the applied recycling technology and that a specific technology can lead to higher migrating amounts of cyclic PET oligomers in the final recycled FCM. In this perspective, although Ubeda et al. reported no differences between vPET and rPET, in fact, the technology (conditions) of the tested rPET was not reported or known [2].

## 4. Conclusions

A QuEChERS clean-up protocol is recommended for sample preparation in order to quantify cyclic PET oligomers that potentially migrate in VOO samples. The proposed method contributes to the analytical challenge of the determination of these substances in complex food matrices, such as edible oils. The method was validated in terms of its linearity, sensitivity, precision and accuracy and proved to be capable of making quantitative statements at target levels of interest. Based on the results, recovery was satisfactory, ranging from 86.6 to 113.0%. Linearity was acceptable, with R^2^ values above 0.99 in all cases, and the method proved to be precise and accurate. Sensitivity was higher for the larger MW oligomers compared to cyclic dimers, an outcome which is in accordance with previous studies and possibly occurred due to compound hydrolysis. VOO samples were stored in vPET and rPET bottles for a year, and the proposed method was used for the evaluation of PET migration. None of the targets were detected in food samples after storage under household conditions. A food simulant for fatty foods (95% *v*/*v* ethanol) stored in a constant temperature incubator at 60 °C for 10 days was found to contain high concentrations of tested compounds (especially the PET trimers), indicating a potential public health concern. However, our results also suggest that the equivalence of the official simulation test method of the EU to the worst-case storage conditions during the food’s shelf life may be challenged. Additionally, abundant linear oligomers, apparently reaction products of the cyclic oligomers with the solvent, were also identified and relatively quantified. In the case of rPET bottles, the total migrating content was significantly higher, indicating that the production technology could be crucial for the safety characteristics of recycled PET bottles. Again, PET trimer oligomers were found to be the most abundant compounds. To the best of our knowledge, this is the first reported method for the quantification of cyclic PET oligomers in VOO and the first fully quantitative method for the determination of PET migration from recycled material.

## Figures and Tables

**Table 1:** Extraction recovery (R%) of target compounds at three concentration levels.

Compound	Concentration Level (μg L^−1^)	Extraction Recovery (R%)
PET dimers	50	86.6
	500	101
	1000	98.0
PET trimers	50	98.5
	500	96.7
	1000	104
PET tetramers	50	111
	500	106
	1000	113
PET pentamers	50	91.3
	500	97.2
	1000	105

**Table 2:** Student’s *t*-test between the calibration curves in VOO and solvent.

Compound	t_exp_	t*_crit_
PET dimers	45.743	2.262
PET trimers	69.083	2.262
PET tetramers	35.339	2.228
PET pentamers	27.303	2.262

* At the 95% confidence level and (n_1_ − 2) + (n_2_ − 2) degrees of freedom.

**Table 3:** Analytical features of the proposed method.

Compound	Molecular Formula	Molecular Ion (*m*/*z*)	Retention Time (min)	Linear Range (μg L^−1^)	Linear Coefficient of Determination (R^2^)	LOD (μg L^−1^)	LOQ (μg L^−1^)
PET dimers	C_20_H_16_O_8_	385.0918	2.5	50–2500	0.998	16.7	50
PET trimers	C_30_H_24_O_12_	577.1346	3.3	10–1000	0.998	3.3	10
PET tetramers	C_40_H_32_O_16_	769.1764	4.1	10–2500	0.996	3.3	10
PET pentamers	C_50_H_40_O_20_	961.2192	4.8	10–1500	0.992	3.3	10

**Table 4:** Intra-day and inter-day precision of assay in spiked virgin olive oil sample.

Compound	Intra-Day (*n* = 6)				Inter-Day (*n* = 3)			
	Added (μg L^−1^)	Found ± S_d_ (μg L^−1^)	S_r_ (%)	E_r_ (%)	Added (μg L^−1^)	Found ± S_d_ (μg L^−1^)	S_r_ (%)	E_r_ (%)
PET dimer	50	56.36 ± 3.27	7.1	12.7	50	58.16 ± 4.32	6.0	16.3
	500	497.0 ± 18.39	3.7	−0.6	500	457.1 ± 43.72	9.6	−8.6
	1500	1600 ± 161.3	10.1	6.7	1500	1580 ± 152.1	9.6	5.3
PET trimer	10	8.45 ± 1.14	13.5	−15.5	10	8.99 ± 0.76	8.5	−10.1
	100	115.2 ± 2.77	2.4	15.1	100	111.7 ± 12.23	10.9	11.7
	500	511.2 ± 15.99	3.1	2.2	500	469.0 ± 39.7	8.5	−6.2
PET tetramer	10	10.64 ± 0.85	8.01	6.3	10	10.58 ± 1.15	10.9	5.9
	500	504.9 ± 75.40	14.9	1.0	500	459.6 ± 58.87	12.8	−8.1
	1500	1520 ± 110.1	7.2	1.3	1500	1565 ± 46.95	3.0	4.4
PET pentamer	10	11.74 ± 0.24	2.0	17.4	10	10.61 ± 1.41	13.3	6.1
	100	93.84 ± 4.13	4.4	−6.2	100	90.94 ± 6.18	6.8	−9.1
	750	765.5 ± 36.57	4.8	2.1	750	734.4 ± 50.09	6.8	−2.1

S_d_, standard deviation; S_r_, relative standard deviation; E_r_, relative error.

**Table 5:** Migrated concentrations (μg L^−1^ ± S_d_) of cyclic oligomers from virgin and recycled PET material in food simulant D2 (ethanol:water 95:5 *v*/*v*).

Oligomers	rPET		vPET	
	Sample No. 1	Sample No. 2	Sample No. 1	Sample No. 2
PET dimer	198.2 ± 10.82	179.0 ± 11.98	42.73 ± 2.17	38.76 ± 1.91
PET trimer	2757 ± 33.61	2950 ± 42.16	1029 ± 71.45	586.9 ± 80.38
PET tetramer	9.32 ± 0.29	8.81 ± 0.01	0.57 ± 0.03	0.51 ± 0.03
PET pentamer	2.57 ± 0.11	2.14 ± 0.06	N/A	N/A

## Data Availability

The data presented in this study are available upon request from the corresponding author.

## Supplementary Materials

The following supporting information can be downloaded at: https://www.mdpi.com/article/10.3390/foods12142739/s1, Figure S1: (A) UHPLC-qTOF-MS extracted ion chromatogram of ionic mass 623.1744 Da of a representative D2 solvent and (B) the corresponding molecular structure identified as H-[TPA-EG]_3_-O-C_2_H_5_; Figure S2: Representative TICs of food simulants D2 from (A) nPET and (B) rPET bottles after their storage in accelerated conditions; Table S1: Tentative quantification results (μg L^−1^) of PET linear oligomers from recycled and virgin PET in D2 food simulants. Linear oligomers were identified based on monoisotopic molecular mass in accordance with [16]) unless otherwise stated.
