# Supplementary material for: Analysis of Migrant Cyclic PET Oligomers in Olive Oil and Food Simulants Using UHPLC-qTOF-MS
Source: Foods. 2023 Jul 19;12(14):2739. doi: 10.3390/foods12142739 (PMC10379823; doi:10.3390/foods12142739)
Supplement: Supplementary file 1 [file foods-12-02739-s001.zip › foods-2479968-supplementary.pdf]

Analysis of PET Cyclic Oligomers Migrated in Olive Oil and Food Simulants Using UHPLC-qTOF-MS

Dimitra Diamantidou [1,2], Emmanouil Tsochatzis [3], Stavros Kalogiannis [2,4,*], Joao Alberto Lopes [5], Georgios Theodoridis [1,2] and Helen Gika [2,6,*]

[1] Laboratory of Analytical Chemistry, Department of Chemistry, Aristotle University of Thessaloniki, 541 24, Thessaloniki, Greece

[2] Biomic_AUTh, Center for Interdisciplinary Research and Innovation (CIRI-AUTH), Balkan Center, B1.4, Thessaloniki, 10th km Thessaloniki-Thermi Rd, P.O. Box 8318, GR 57001

[3] Department of Food Science, iFOOD, Centre for Innovative Food Research, Aarhus University, Agro Food Park 48, Aarhus N, 8200, Denmark.Biomic_AUTh, Center for Interdisciplinary Research and Innovation

[4] Department of Nutritional Sciences and Dietetics, International Hellenic University, Thessaloniki, GR-574 00, Greece

[5] European Innovation Council and SMEs Executive Agency (EISMEA), Brussels, Belgium

[6] School of Medicine, Aristotle University of Thessaloniki, Thessaloniki, 54124, Greece

* Correspondence: SK: kalogian@ihu.gr; HG: gkikae@auth.gr

Supplementary information

Table S1. Tentative quantification results (μg L^{-1}) of PET linear oligomers from recycled and virgin PET in food simulants D2. Linear oligomers were identified based on monoisotopic molecular mass in accordance with Tsochatzis et al. (2020), unless otherwise stated.

Linear oligomer code	Precursor ion (m/z)	Retention time (min)	rPET		vPET	
			Sample no.1	Sample no.2	Sample no.1	Sample no.2
H-$[TPA\text{-}EG]_2$-H	387.1924	2.5	39.36±0.21	35.51±1.23	113.4±7.12	102.2±5.32
H-$[TPA\text{-}EG]_2$-OH	403.1016	1.8	41.33±2.31	40.81±5.67	61.14±3.98	47.22±3.45
H-$[TPA\text{-}EG]_3$-OH	594.1593	3.2	3953±32.34	2845±54.43	738.2±18.56	378.7±8.88
H-$[TPA\text{-}EG]_3$-OC_2H_5*	623.1744	3.1	4282±56.21	3994±39.76	822.4±11.56	645.1±9.67
H-$[TPA\text{-}EG]_3$-EG-OH	639.1697	2.2	83.52±6.54	80.78±4.87	24.98±1.95	19.17±0.65

* Identification based on monoisotopic molecular mass.

(A)

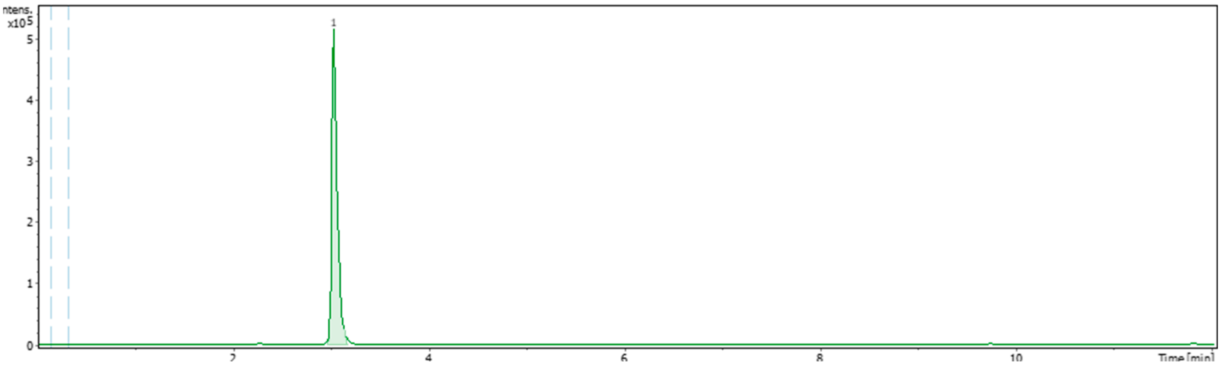

(B)

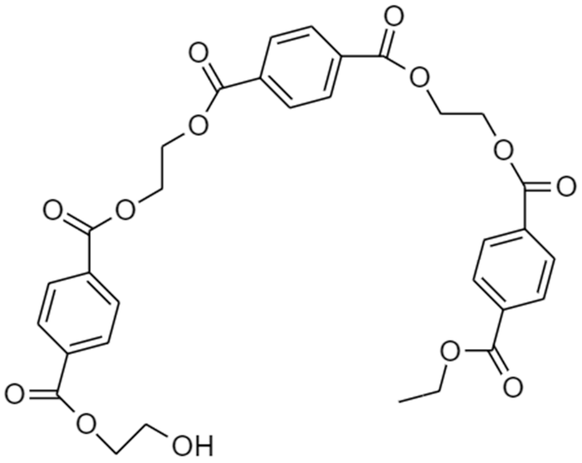

Figure S1. (A) UHPLC-qTOF-MS extracted ion chromatogram of ionic mass 623.1744 Da of a representative D2 solvent and (B) the corresponding molecular structure identified as H-[TPA-EG]$_3$-O-C_2H_5.

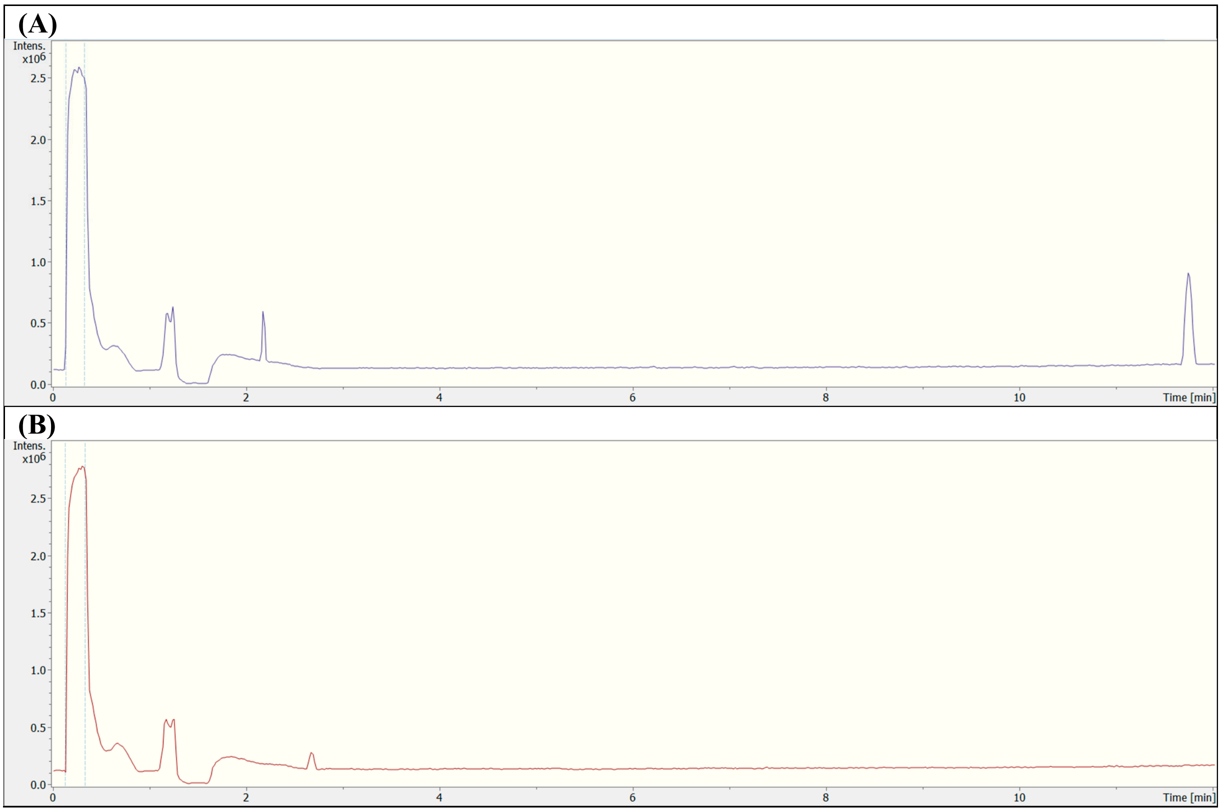

Figure S2. Representative TICs of food simulants D2 from (A) nPET and (B) rPET bottles after their storage in accelerated conditions.
